# Impact of Underlying Pulmonary Arterial Hypertension on the In-Hospital Outcomes of Patients Admitted Due to Atrial Fibrillation, Atrial Flutter, Ventricular Tachycardia, or First Myocardial Infarction: A Nationwide Analysis (2016–2020)

**DOI:** 10.7759/cureus.80332

**Published:** 2025-03-10

**Authors:** Christian Siochi, Wilmer F Cervantes, Geovanny F Cervantes, Bolaji Durodola, Lourdes Villarrubia Varela, Danny Segura Torres, Stephen Jesmajian

**Affiliations:** 1 Internal Medicine, Montefiore New Rochelle Hospital, Albert Einstein College of Medicine, New Rochelle, USA

**Keywords:** atrial fibrillation, atrial flutter, myocardial infarction, pulmonary artery hypertension, ventricular tachycardia

## Abstract

Background

Pulmonary Arterial Hypertension (PAH) is known to impact heart disease outcomes. In this analysis, we aim to analyze the impact of atrial fibrillation/atrial flutter (AF), ventricular tachycardia (VT), or a first myocardial infarction (MI) episode on patients with PAH. This will improve understanding of the clinical impact of underlying PAH in patients who develop these conditions to create a risk stratification process and possibly guidelines regarding their management.

Methods

In this National Inpatient Sample Database (2016-2020) analysis, patients admitted with a primary diagnosis of AF, VT or first MI episode, with or without a secondary diagnosis of PAH were identified using International Classification of Diseases, Tenth Revision, Clinical Modification (ICD-10-CM) codes. The primary outcome was mortality. Secondary outcomes included the length of stay, resource utilization, and the necessity for endotracheal intubation and cardiac assistance devices. Univariate analysis included hospital-level and patient baseline characteristics such as age, gender, race, Charlson comorbidity index, hospital location, size, region, teaching status, and insurance. Baseline characteristics with p-value <0.2 were considered significant and adjusted in a multivariate analysis. Data was statistically significant if p-value <0.05.

Results

From 2016 to 2020, out of the adults admitted for AF (n=2,292,194), VT (n=241,225), and first MI (n=2,567,159), those who had PAH were 119,095, 12,470, and 79,895, respectively. Appropriate diagnosis and classification of PAH is essential for identifying the possible complications associated with this condition. Patients admitted for AF with a secondary diagnosis of PAH had a higher mortality risk (OR 1.22; 95% CI 1.09-1.37; p=0.001), longer length of stay in days (regression coefficient 0.89; 95% CI 0.82-0.96; p<0.001), greater resource utilization in dollars (regression coefficient 11510.71; 95% CI 10120.46-12900.97; p<0.001) and more endotracheal intubations (OR 1.69; 95% CI 1.46-1.96; p<0.001), but showed no difference on cardioversions (OR 1.10; 95% CI 0.94-1.29; p=0.241). Patients admitted due to VT with a secondary diagnosis of PAH also had a higher mortality risk (OR 1.39; 95% CI 1.13-1.71; p=0.002), greater length of stay in days (regression coefficient 1.22; 95% CI 0.87-1.58; p<0.001), higher resource utilization (regression coefficient 25332.61; 95% CI 16305.56-34359.66; p<0.001), and more endotracheal intubations (OR 1.37; 95% CI 1.11-1.68; p=0.003) and cardioversions (OR 1.13; 95% CI 1.25-1.36; p<0.001). Adjusted outcomes showed that patients with PAH admitted for first MI had an increased in-hospital mortality risk (OR 1.11; 95% CI 1.03-1.2; p=0.006), length of stay (regression coefficient 1.35; 95% CI 1.21-1.48; p<0.001), hospital charges (regression coefficient 23050.94; 95% CI 18952.86-27149.03; p<0.001), and rate of intubation (OR 1.24; 95% CI 1.14-1.35; p<0.001).

Conclusion

Our investigation shows a clear detrimental trend in patients that are admitted to the hospital with AF, VT, and first MI along with an underlying history of PAH. Compared to those without a history of this pulmonary condition, such patients have an increased mortality rate as well as an increased length of hospital stay, higher hospital charges and some other in-hospital complications. More studies are necessary to assess the impact of specific therapies for PAH in order to evaluate the effect on outcomes.

## Introduction

Pulmonary arterial hypertension (PAH) is a progressive and debilitating ailment that leads to increased morbidity and mortality due to worsening right ventricular dysfunction and pulmonary vasculopathy [1]. Globally, the prevalence of pulmonary hypertension is 1% and its etiology varies depending on the functional classification [2]. Studies have found that in the United States, high-risk PAH patients have an incredibly high mortality rate in addition to increased direct (i.e. resource utilization) and indirect (loss of productivity and earnings) healthcare costs [3,4]. Consequently, early detection and treatment of PAH remain paramount to mitigating its detrimental consequences. 

Atrial fibrillation (AF) is the most prevalent arrhythmia worldwide and in the United States. Its prevalence in the United States is projected to increase by 2.5-fold and reach 7.5 million cases by 2050 [5]. AF is depicted as an enormous economic and public challenge due to the increased number of hospitalizations and all-cause mortality attributed to it [6]. 

Interestingly, multiple comorbidities such as arrhythmias and ischemic heart disease have been associated with PAH at the time of diagnosis [7]. Unfortunately, the presence of comorbidities can delay the diagnosis and early detection of PAH since they mask its symptomatology. This can lead to a false assessment of disease progression and therapy response [7]. 

Studies have documented the incidence of atrial arrhythmias (AA) in patients with PAH. Furthermore, such patients present with clinical complications and signs of right-side heart failure [8,9]. The hallmark of the pathophysiology in patients with AA and PAH is the enlargement of the right atrium as a result of advanced disease and the presence of right-side heart failure. The increase in the chamber size and right atrial pressure predispose the patient to the development of AF [10]. Additionally, the chronic right atrial pressure, stretch and overload, paired with chronic hypoxia, promote atrial fibrosis, which in turn also increases the development of AF [11]. On the other hand, a low incidence of ventricular arrhythmias, such as ventricular tachycardia (VT) and ventricular fibrillation has been noted in patients with PAH as compared to those with primarily left ventricular failure [12]. Multiple studies have shown a relationship between supraventricular arrhythmias and clinical deterioration in patients with PAH, leading to an increased number of hospitalizations, ICU admissions, and use of vasopressors [13]. 

Another important relationship between the heart and the lungs relates to the interaction of pressures in the vasculature. It is thought that the development of myocardial infarction (MI) can be explained by the mechanical pressure that results from high pulmonary venous pressure in people with underlying ischemic cardiomyopathy and left ventricular systolic dysfunction [14].

Aim of the study

PAH is known to impact arrhythmia outcomes based on the reported pathophysiology and the hemodynamic changes that occur due to the elevated mean pulmonary artery pressure that is triggered by both these conditions. However, there is a lack of data assessing the degree of this impact, and as a result, there is limited data regarding the associated rate of mortality and hospitalization. This study aims to elucidate the burden that PAH levies on patients admitted due to arrhythmias as well as MI. This article is a compilation of two poster contributions previously presented at the American College of Cardiology (ACC) 2024 Annual Conference on April 7, 2024 and American College of Chest Physicians (CHEST) 2024 Annual Meeting on October 6-9, 2024.

## Materials and methods

Study design 

This was a retrospective cohort study that used discharge data from the National Inpatient Sample (NIS), the Healthcare Cost and Utilization Project (HCUP), and the Agency for Healthcare Research and Quality from 2016 to 2020. 

Inclusion criteria 

Adult patients (≥18 years) with AF, VT, first ST-segment elevation MI (STEMI), or first non-ST-segment elevation MI (NSTEMI) and with or without a secondary diagnosis of PAH were identified using the International Classification of Disease, Tenth Edition, Procedure Coding System (ICD-10-PCS) and the International Classification of Disease, Tenth Edition, Clinical Modification (ICD-10-CM) codes.

Ethical considerations 

The data from the NIS-HCUP database is publicly available, deidentified, and exempt from institutional review board approval. The need for informed consent was waived. 

Outcome measures 

The primary outcome of interest was in-hospital mortality, while secondary outcomes included length of stay in days, total hospital charges in dollars, rate of endotracheal intubation, and rate of cardioversion. 

Statistical analysis 

This analysis used a CI of 95% and a p-value <0.05 as statistically significant. Continuous variables were examined through the calculation of means accompanied by standard deviations (SDs) or medians along with interquartile ranges (IQRs) in the case of normally distributed and skewed data, respectively. Descriptive statistics incorporating frequencies and percentages were employed for the analysis of categorical variables. The patient and hospital-level baseline characteristics, and in-hospital outcomes were compared between patients with a primary diagnosis of AF, VT or first MI, aged >18 years, with or without a secondary diagnosis of PAH using the Pearson χ2 test for categorical variables and the independent sample t-test for continuous variables.

To calculate the unadjusted and adjusted ORs for in-hospital clinical outcomes, univariate and multivariate logistic regressions were used. First, a univariate analysis was used to identify patient and hospital-level baseline characteristics with p value <0.2. A multivariate analysis was then done to adjust for these significant baseline characteristics and to account for potential confounding factors. All analyses were conducted using STATA version 13 (StataCorp LLC, College Station, TX, US). 

## Results

The baseline patient characteristics, including race and Charlson comorbidity index, and hospital-level characteristics for the AF (Table 1), VT (Table 2), and first MI (Table 3) cohorts are shown below.

**Table 1:** Baseline characteristics of patients with atrial fibrillation (AF) The percentage of patients with a primary diagnosis of AF, per baseline patient and hospital-level characteristics, with or without a secondary diagnosis of PAH. To account for the potential confounding factors, a multivariate regression model was later adjusted for patient and hospital-level baseline characteristics with a p-value <0.2. PAH: Pulmonary arterial hypertension

Baseline characteristics of patients with AF		Without PAH (% of patients; total n=2,173,099)	With PAH (% of patients; total n=119,095)	Complete cohort (% of patients; total n=2,292,194)	p-value
Sex	Male	51.74	38	51.03	<0.01
	Female	48.26	62	48.97	
Race	White	81.92	78.49	81.74	<0.01
	Black	8.27	12.01	8.46	
	Hispanic	5.88	5.4	5.85	
	Asian	1.5	1.77	1.51	
	Native American	0.37	0.36	0.37	
	Other	2.07	1.96	2.06	
Charlson comorbidity index	0	23.08	6.98	22.24	<0.01
	1	25.85	20.15	25.56	
	2	19.48	22.37	19.63	
	>3	31.59	50.5	32.57	
Median income	<49999	27.41	28.05	27.44	0.001
	50000-64999	27.25	28.19	27.3	
	65000-85.999	24.73	24.11	24.7	
	>86000	20.61	19.64	20.56	
Type of insurance	Medicare	69.79	78.67	70.25	<0.01
	Medicaid	6.43	6.19	6.42	
	Private insurance	21.25	13.4	20.84	
	Self pay	2.53	1.74	2.49	
Hospital region	Northeast	19.61	19.4	19.6	<0.01
	Midwest	24.2	24.68	24.22	
	South	40.98	39.19	40.89	
	West	15.22	16.73	15.29	
Hospital bed size	Small	21	21.96	21.05	0.12
	Medium	30.01	29.46	29.98	
	Large	48.99	48.59	48.97	
Hospital location	Rural	10.63	9.39	10.58	<0.01
	Urban	89.37	90.61	89.42	
Hospital teaching status	No	34.38	29.48	34.16	<0.01
	Yes	65.62	70.52	65.84	

**Table 2:** Baseline characteristics of patients with ventricular tachycardia (VT) The percentage of patients with a primary diagnosis of VT, per baseline patient and hospital-level characteristics, with or without a secondary diagnosis of PAH. To account for the potential confounding factors, a multivariate regression model was later adjusted for patient and hospital-level baseline characteristics with a p-value <0.2. PAH: Pulmonary arterial hypertension

Baseline characteristics of patients with VT		Without PAH (% of patients; total n=228,755)	With PAH (% of patients; total n=12,470)	Complete cohort (% of patients; total n=241,225)	p-value
Sex	Male	74.84	70.97	76.64	<0.001
	Female	25.16	29.03	25.36	
Race	White	76.43	69.33	76.06	<0.001
	Black	13.24	20.23	13.61	
	Hispanic	5.74	5.8	5.74	
	Asian	1.87	1.97	1.88	
	Native American	0.47	0.41	0.46	
	Other	2.25	2.26	2.25	
Charlson comorbidity index	0	9.73	1.68	9.31	<0.001
	1	14.86	8.06	14.51	
	2	19.49	15.76	1.93	
	>3	55.93	7.45	56.89	
Median income	<49999	27.21	27.22	27.21	0.0776
	50000-64999	26.36	28.12	26.45	
	65000-85.999	24.84	25.14	24.86	
	>86000	21.59	19.52	21.48	
Type of insurance	Medicare	64.86	74.01	65.34	<0.001
	Medicaid	8.46	0.79	8.43	
	Private insurance	24.48	16.86	24.08	
	Self pay	2.21	1.23	2.16	
Hospital region	Northeast	20.65	20.09	20.62	0.014
	Midwest	23.45	26.38	2.36	
	South	3.89	35.89	38.74	
	West	0.17	17.64	17.04	
Hospital bed size	Small	1.45	15.56	14.56	0.1067
	Medium	2.69	24.86	26.79	
	Large	5.86	59.58	58.65	
Hospital location	Rural	5.31	4.03	5.25	0.0234
	Urban	94.69	95.97	94.75	
Hospital teaching status	No	2.36	17.61	23.33	<0.001
	Yes	7.64	82.39	76.67	

**Table 3:** Baseline characteristics of patients with the first myocardial infarction (MI) The percentage of patients with a primary diagnosis of first MI, per baseline patient and hospital-level characteristics, with or without a secondary diagnosis of PAH. To account for the potential confounding factors, a multivariate regression model was later adjusted for patient and hospital-level baseline characteristics with a p-value <0.2. PAH: Pulmonary arterial hypertension

Baseline characteristics of patients with first MI		Without PAH (% of patients; total n=2,487,263)	With PAH (% of patients; total n=79,895)	Complete cohort (% of patients; total n=2,567,159)	p-value
Sex	Male	63.23	56.16	62.07	<0.01
	Female	36.77	43.84	37.93	
Race	White	71.82	80.72	73.29	<0.01
	Black	11.48	9.91	11.22	
	Hispanic	9.6	5.36	8.9	
	Asian	3.19	1.34	2.88	
	Native American	0.59	0.53	0.58	
	Other	3.33	2.15	3.13	
Charlson comorbidity index	0	0	0	0	<0.01
	1	30.26	0	25.29	
	2	27.15	16.77	25.44	
	> 3	42.58	83.23	49.27	
Median income	<49999	29.13	37.42	30.5	<0.01
	50000-64999	27.34	29.5	27.7	
	65000-85.999	23.95	20.54	23.39	
	>86000	19.57	12.54	18.42	
Type of insurance	Medicare	54.65	72.36	57.57	<0.01
	Medicaid	9.58	10.17	9.68	
	Private insurance	30.34	14.62	27.75	
	Self pay	5.43	2.84	5	
Hospital region	Northeast	17.77	15.94	17.47	<0.01
	Midwest	21.61	24.56	22.1	
	South	41.11	44.39	41.65	
	West	19.51	15.1	18.78	
Hospital bed size	Small	18.13	18.62	18.21	<0.01
	Medium	30.31	31.36	30.48	
	Large	51.56	50.03	51.31	
Hospital location	Rural	7.16	10.34	7.69	<0.01
	Urban	92.84	89.66	92.31	
Hospital teaching status	No	30.31	34.39	30.99	<0.01
	Yes	69.69	65.61	69.01	

In the United States, between 2016 and 2020, of the 2,292,194 adults admitted due to AF, 119,095 had PAH and 2,173,099 did not. Similarly, of the 241,225 patients admitted due to VT, 12,470 had PAH and 228,755 did not, and of the 2,567,159 adults admitted for a first MI episode, 79,895 had PAH and 2,487,264 did not. 

The mean age of the AF population with PAH was 73.71 years, while for those without PAH was 70.42 years. Similarly, the mean age of VT population with and without PAH was 68.44 and 66.37 years, respectively. The mean age of the patient population with first MI with and without PAH was 69.74 and 66.01 years, respectively 

The majority of patients included in this study were white, male, and with a Charlson comorbidity index greater than three. The percentage of patients with a primary diagnosis of AF, VT or first MI, per baseline patient and hospital-level characteristics, with or without a secondary diagnosis of PAH are given in Tables 1-3. 

Primary outcome 

Adjusted outcomes showed that patients with a secondary diagnosis of PAH admitted due to AF, VT, or first MI had an increased in-patient mortality risk of 22% (OR 1.22; 95% CI 1.09-1.37; p=0.001), 39% (OR 1.39; 95% CI 1.13-1.71; p=0.002), and 11% (OR 1.11; 95% CI 1.03-1.2; p=0.006) respectively (Table 4).

**Table 4:** Primary outcome measure across cohorts Adjusted odds ratio for the mortality outcome of patients admitted for AF, VT, first STEMI or first NSTEMI, with a secondary diagnosis of PAH compared to those without PAH. This analysis used a confidence interval of 95% and a p-value <0.05 as statistically significant. AF: Atrial fibrillation/atrial flutter, VT: Ventricular tachycardia, STEMI: ST-segment elevation myocardial infarct, NSTEM: Non-ST segment elevation myocardial infarction, MI: Myocardial infarction, PAH: Pulmonary arterial hypertension

Primary outcome adjusted for variables with p<0.2						
In-patient mortality	Odds ratio	Std. error	t	p-value	95% Confidence Interval	
AF	1.221	0.072	3.36	0.001	1.087	1.372
VT	1.389	0.149	3.06	0.002	1.126	1.714
First STEMI	1.29	0.1	3.17	0.002	1.1	1.51
First NSTEMI	1.23	0.06	4.5	<0.001	1.12	1.34
Total first MI	1.11	0.04	2.76	0.006	1.03	1.2

Secondary outcomes 

Adjusted outcomes showed that patients with a secondary diagnosis of PAH admitted due to AF, VT, or first MI had an increased length of hospital stay in days and higher hospital charges in dollars (Table 5).

**Table 5:** Secondary outcomes across all cohorts Adjusted regression coefficients for the outcomes of length of hospital stay and total hospital charges in case of patients admitted for AF, VT, first STEMI or first NSTEMI with a secondary diagnosis of PAH. This analysis used a confidence interval of 95% and a p-value <0.05 as statistically significant. AF: Atrial fibrillation/atrial flutter, VT: Ventricular tachycardia, STEMI: ST-segment elevation myocardial infarction, NSTEMI: Non-ST segment elevation myocardial infarction, MI: Myocardial infarction, PAH: Pulmonary arterial hypertension

Secondary outcomes adjusted for variables with p < 0.2						
Length of stay (days)	Coefficient	Std. error	t	p-value	95% Confidence Interval	
AF	0.886	0.035	24.99	<0.001	0.816	0.955
VT	1.224	0.183	6.68	<0.001	0.865	1.583
First STEMI	1.7	0.19	8.98	<0.001	1.33	2.07
First NSTEMI	1.3	0.07	18.23	<0.001	1.16	1.44
Total first MI	1.35	0.07	19.84	<0.001	1.21	1.48
Total hospital charges (dollars)	Coefficient	Std. error	t	p-value	95% Confidence Interval	
AF	11510.71	709.283	16.23	<0.001	10120.46	12900.97
VT	25332.61	4605.427	5.5	<0.001	16305.56	34359.66
First STEMI	43477.92	6924.185	6.28	<0.001	29905.91	57049.92
First NSTEMI	22404.59	1924.04	11.64	<0.001	18633.3	26175.87
Total first MI	23050.94	2090.768	11.03	<0.001	18952.86	27149.03

As detailed in Table 6, patients admitted due to AF with a secondary diagnosis of PAH had increased endotracheal intubations (OR 1.69; 95% CI 1.46-1.96; p<0.001) with no difference in cardioversions (OR 1.10; 95% CI 0.94-1.29; p=0.241). Patients admitted due to VT with a secondary diagnosis of PAH had increased endotracheal intubations (OR 1.37; 95% CI 1.11-1.68; p=0.003) and cardioversions (OR 1.13; 95% CI 1.25-1.36; p<0.001). Patients admitted due to first MI with a secondary diagnosis of PAH had an increased rate of intubation (OR 1.24; 95% CI 1.14-1.35; p<0.001).

**Table 6:** Additional secondary outcomes across cohorts Adjusted odds ratio for endotracheal intubation and cardioversion outcomes of patients admitted for AF, VT, first STEMI or first NSTEMI with a secondary diagnosis of PAH. This study used a confidence interval (CI) of 95% and a p-value <0.05 as statistically significant in its analysis. AF: Atrial fibrillation/atrial flutter, VT: Ventricular tachycardia, STEMI: ST-segment elevation myocardial infarction; NSTEMI: Non-ST segment elevation myocardial infarction, MI: Myocardial infarction, PAH: Pulmonary arterial hypertension

Additional secondary outcomes adjusted for variables with p <0.2						
Endotracheal intubation	Odds ratio	Std. error	t	p-value	95% Confidence Interval	
AF	1.692	0.126	7.08	<0.001	1.463	1.957
VT	1.366	0.144	2.96	0.003	1.112	1.68
First STEMI	1.53	0.13	5.19	<0.001	1.31	1.8
First NSTEMI	1.34	0.07	5.79	<0.001	1.22	1.48
Total first MI	1.24	0.05	4.9	<0.001	1.14	1.35
Cardioversion	Odds ratio	Std. error	t	p-value	95% Confidence Interval	
AF	1.099	0.089	1.17	0.241	0.938	1.287
VT	1.3	0.028	12.24	<0.001	1.246	1.355

The findings obtained during this investigation demonstrated the importance of the concomitant presentation of PAH with underlying cardiac conditions and its implication on clinical outcomes. It provided an opportunity to better conceptualize the burden of PAH in terms of measurable outcomes, including mortality. 

## Discussion

In this study, the group of patients with a past medical history of PAH exhibited a significantly higher number of complications when they were admitted with AF, as well as first onset of MI. Other parameters, such as the length of hospital stay, money spent, and the need for intubation and cardioversion, were also more of a burden for those with an underlying history of PAH. This analysis revealed that PAH has a negative impact on the outcomes of patients admitted with certain cardiovascular events. Further studies are needed to elucidate the need for specific treatments based on the severity of the underlying disease [15]. 

The primary outcome of interest in this study was in-patient mortality of the patients with a history of PAH who presented with new-onset MI, heart failure, and two of the common arrhythmias. A possible explanation for this phenomenon may relate to the presence of other metabolic and inflammatory states in addition to other pre-existing comorbidities. 

In the case of MI, the interaction with pressures in left-side heart disease can increase right ventricular systolic pressure. This also occurs in PAH resulting in worsened hemodynamics [16,17]. It has also been associated with increased mortality in the setting of abnormalities in left ventricular function and contractility [17]. In a retrospective study, pulmonary hypertension was used as a prognostic parameter in the mortality outcome of patients admitted with MI. The researchers presented a nomogram with three additional risk factors, including abnormal left ventricular ejection fraction (LVEF), creatinine kinase-MB (CK-MB), and pro-B-type natriuretic peptide (Pro-BNP). Essentially, the study showed that the lab results could be used to predict one-year mortality risk [18]. Additionally, the presence of a larger left atrial volume and moderate to severe mitral regurgitation, which are seen with elevated systolic arterial pressure, could explain the increased risk of all-cause mortality in patients treated with percutaneous coronary intervention after STEMI, as it can further worsen right-sided pressures [19,20]. 

Heart failure is another common hospital presentation, whose outcomes seem to be influenced by the presence of an elevated pulmonary arterial pressure. A study proposed that the systemic changes that occur in PAH, including reduced organ perfusion, contribute to the susceptibility to organ damage and other complications. The maladaptive systemic changes such as neurohormonal imbalance and oxidative stress lead to water and salt retention as well as vascular congestion and result in reduced cardiac output [16]. A review of the data in a study revealed that there is an increase in morbidity and mortality when these two pathologies coexist. The researchers explained the pathophysiology in the mechanism of the postcapillary, which leads to an increased pulmonary arterial pressure due to an elevated left atrial pressure. This contributes to aberrancies in the hemodynamic changes in heart failure, which is propagated by the underlying pulmonary hypertension [16]. Another study that explored the prognostic implications of reactive pulmonary hypertension with a background of passive pulmonary hypertension demonstrated an increase in mortality rates in patients that presented with acute decompensated heart failure. Multivariate adjustments were applied and certain parameters, including pulmonary vascular resistance, wedge pressure, and arterial pressure, were compared in three different groups. The investigators concluded that when compared to no pulmonary or passive pulmonary hypertension, reactive pulmonary hypertension (common in patients with acute exacerbation of heart failure after treatment with diuretics and vasodilators) was an independent predictor of death (95% CI 1.7-4.7, P=0.0001) [21,22]. 

In AF, several mechanisms have been proposed for the complicated outcomes regarding long-term survival. A common explanation pertains to the loss of appropriate or regular contraction of the heart impeding normal ventricular function, which affects cardiac output [23]. A study that involved patients with AF used an invasive test to induce various pacing modes. The researchers observed a decreased cardiac output (4.4 +/- 1.6 vs. 5.2 +/- 2.4 liters/min, p<0.01), increased pulmonary capillary wedge pressure (17 +/- 7 vs. 14 +/- 6 mmHg, p<0.002), and increased right atrial pressure (10 +/- 6 vs. 8 +/- 4 mmHg, p<0.05) in the group with an irregular sequence of RR intervals [23]. A different study assessed mortality in patients with AA with an underlying history of PAH. It specifically focused on those with idiopathic and systemic sclerosis related PAH. Some of the explanations for the higher mortality observed in those with AA were related to an increased right atrial and wedge pressure as well as higher N-terminal (NT)-pro-BNP and prevalence of thyroid disease. Though not statistically significant (log rank P=0.323), the researchers did observe a higher mortality in patients with AA with underlying PAH [13]. 

Importantly, when evaluating pulmonary hypertension with other coexisting cardiovascular diseases, other implications also exist. In addition to an increase in poor outcomes and the resulting increased mortality rates, there is also an increase in the length of hospital stay and total cost as well as the need for mechanical ventilation. The length of hospital stay and expenses seem to be greater in complicated cases. Such cases might necessitate certain interventions, like percutaneous coronary intervention (PCI), which could also increase the total costs [15]. With regards to the need for mechanical ventilation, there is currently no consensus about the pathophysiological basis, but some studies suggest that pulmonary hypertension has an effect on the skeletal muscle and the diaphragm [15].

Understanding and acknowledging the complexity of PAH when it co-exists with other cardiac conditions provides an opportunity for more research. A review of data shows that the management of patients with AF as well as VT and acute coronary syndrome presents is more of a challenge with underlying PAH. This is primarily explained by the changes in hemodynamics that take place in the right side of the heart. These can be exacerbated and exist as a positive feedback loop in the setting of arrhythmias and also ischemia. Additional studies could provide more information about the influence of other underlying medical problems as well possible relevant differences in management and outcomes based on the different subtypes of AF and pulmonary hypertension. 

Study limitations 

Our study has some limitations. It was conducted using the NIS, which is the largest database that collects hospital stay information. In comparison to electronic health records, the NIS lacks detailed and in-depth data. The use of the NIS relies on the ability of the hospitals to formulate an accurate diagnosis and the use precise procedure codes. As a result, misclassification can occur when there is a variability in procedure codes, for example, while using ICD-10-CM codes. Another possible source of limitation, due to the administrative nature of the database, is the lack of differentiation in the severity of PAH. Similarly, there was no information available on the medications that may have been administered for the observed arrhythmias and MI. This was especially true when we analyzed the secondary outcomes. 

Importantly, the primary cause of death is not stated in the NIS, and hence, all-cause mortality or in-patient mortality was presented instead. This was a retrospective observational study and there was no controlled randomization that took place to study treatment across the different groups. Thus, prospective studies would be of great value to delineate whether certain interventions would affect the desired outcome in addition to addressing some of the aforementioned limitations. Nevertheless, this study provides important data that aims to understand the burden of PAH on patients admitted with certain cardiac comorbidities. Moreover, it contributes toward the relationship of PAH with certain arrhythmias and MI and its implication on secondary outcomes such as the length of stay, hospital outcomes, and treatment. Furthermore, the large sample size in this investigation certainly improves the statistical significance of our results. Classification of pulmonary hypertension may differ as procedure codes may very depending on the institution, providers or tools available for making a diagnosis. As a result, evaluation of cardiac outcomes in the presence of PAH may be influenced by possible misclassification. Similarly, the severity of PAH is another factor that can impact outcomes, and it can influence primary and secondary outcomes differently. This impact is generally more negative with advanced disease. Our study also did not account for patients who were on therapy for their underlying PAH or if they were regularly being followed up regarding their disease. Hence, we cannot compare if these patients may have had different outcomes to compared to those who were not undergoing treatment or being followed up. Moreover, the specific subtypes of AF were not studied separately as they were not categorized. 

Additionally, outcomes can vary if medical problems or complications such as an infection or respiratory failure co-exist, especially when analyzing mortality. As with many other medical diagnoses, medication non-adherence also poses a risk for the development of serious complications. Thus, data on medication compliance can further help elucidate its influence on outcomes and aid in establishing preventive measures.

## Conclusions

A past medical history of PAH in a patient presenting to the hospital with common cardiac complications, namely heart failure, atrial arrhythmias and MI appears to be a common occurrence, leading to poor clinical outcomes. This aspect has not been evaluated enough. This investigation revealed the big impact that PAH has on patients admitted with the aforementioned cardiac complications. There is a definite need for early detection and evaluation of possible treatment modalities based on the type of PAH. This, in turn, can potentially delineate the effects of treatment and improve the management of comorbid arrhythmias and ischemic heart disease in patients with PAH.
